# Genomic diversity and functional insights of carbapenem-resistant Klebsiella pneumoniae revealed by centroid coding sequences analysis

**DOI:** 10.1099/mgen.0.001457

**Published:** 2025-07-25

**Authors:** Jianghong Cao, Dongdong Xue, Dan Gao, Xiaoyang Li, Jinlin Guo, Peng Dai, Gangli Zhang

**Affiliations:** 1 The Intensive Care Unit Department of Shanxi Provincial People's Hospital, Shanxi Medical University, Taiyuan, 030001, PR China; 2 The Pharmacy Department of Shanxi Provincial People's Hospital, Shanxi Medical University, Taiyuan, 030001, PR China; 3 The Hepato-Pancreatic-Biliary Surgery Department of Shanxi Provincial People's Hospital, Shanxi Medical University, Taiyuan, 030001, PR China; 4 The Neurosurgery Department of Shanxi Provincial People's Hospital, Shanxi Medical University, Taiyuan, 030001, PR China

**Keywords:** carbapenem resistance, carbapenem-resistant, classification, *K. pneumoniae*, *Klebsiella pneumoniae*, sequence type, virulence factor

## Abstract

*Klebsiella pneumoniae* is a primary bacterial pathogen responsible for various human infections with the emergence of carbapenem-resistant *K. pneumoniae* (CRKP) as a significant clinical challenge. Although previous studies have classified *K. pneumoniae* based on homology of conserved genes, the biological implications of these classifications remain unclear. In this study, we extracted 47,222 centroid coding sequences (CDSs), representative sequences selected from clusters of highly similar CDSs, from 3,592 complete genomes and analysed the genomic characteristics of 4,458 CRKP strains using these centroid CDSs. This approach revealed substantial genomic diversity among CRKP strains, with classification results largely aligning with the multilocus sequence typing (MLST) method. Associations between centroid CDSs and MLST sequence types (STs) were identified, and the biological functions enriched in each ST including those linked to resistance and virulence mechanisms were highlighted. Moreover, the coexistence of CDSs annotated as antibiotic resistance and virulence genes was observed, particularly between a group of carbapenem resistance genes and those encoding proteins for type I and III fimbriae, efflux pumps, type II secretion system and siderophore-mediated iron acquisition. These findings provide an alternative approach for classifying CRKP strains, offering insights into their genomic characteristics.

**Impact Statement**

This study presents a novel method for classifying carbapenem-resistant *Klebsiella pneumoniae* (CRKP) strains based on centroid coding sequences (CDSs) extracted from complete genomes, offering a new approach to understanding the genetic diversity and biological implications of CRKP. By analysing 47,222 centroid CDSs from 3,592 genomes, the study reveals significant genomic variability within CRKP strains, highlighting associations between specific sequence types and key resistance and virulence mechanisms. Additionally, the study demonstrates the coexistence of specific antibiotic resistance and virulence genes, although the biological significance of this coexistence warrants further investigation. In total, these findings enhance our understanding of the genomic landscape of CRKP strains.

## Data Summary

Complete genomes of *Klebsiella pneumoniae* strains were downloaded from the NCBI genome database (https://www.ncbi.nlm.nih.gov/datasets/genome/). Raw genome sequences of carbapenem-resistant *K. pneumoniae* strains were downloaded from the NCBI SRA database (https://www.ncbi.nlm.nih.gov/sra). All sample IDs are shown in Dataset S1 (available in the online Supplementary Material).

## Introduction

*Klebsiella pneumoniae* is a prevalent bacterial pathogen leading to various human diseases, including but not limited to pneumonia, urinary tract infections, bloodstream infections and wound infections, with a particularly high risk in immunocompromised individuals [1]. The emergence of carbapenem-resistant *K. pneumoniae* (CRKP) has heightened its clinical significance, contributing to higher morbidity, mortality and treatment costs [2]. Furthermore, the rapid rise in carbapenem resistance in *K. pneumoniae* in China, from 2.9% in 2005 to 26.8% in 2019, is a noteworthy trend. Understanding the genomic features underlying the resistance and virulence mechanisms of *K. pneumoniae* is crucial for combating this growing threat.

The primary mechanism of carbapenem resistance in *K. pneumoniae* is the production of carbapenemases, enzymes that degrade carbapenems, e.g. KPC, NDM, OXA-48, IMP and VIM enzymes [2]. Another mechanism is the decreased expression or loss of outer membrane proteins, e.g. OmpK36 and OmpK37, which reduces the ability to take up carbapenems. Additionally, the overexpression of extended-spectrum *β*-lactamases and AmpC cephalosporinases, e.g. TEM, SHV, CTX-M and AmpC, contributes to resistance by breaking down *β*-lactams, including carbapenems. Moreover, efflux pumps, e.g. AcrB and KexD, transport antibiotics out of the bacterial cell, thereby decreasing antibiotic effectiveness.

In addition to carbapenem resistance, hypervirulence presents a critical clinical challenge, contributing to severe health complications. Carbapenem-resistant and hypervirulent *K. pneumoniae* strains have emerged, with clinical evidence suggesting that carbapenem resistance is more often acquired before hypervirulence rather than the reverse [3,4]. Although genomic sequencing has identified associations between carbapenem resistance and hypervirulence genes, these findings are observational, and establishing a direct causal relationship with clinical outcomes requires further investigation [5,6]. Nonetheless, the potential interaction between carbapenem resistance and hypervirulence genes remains a pivotal question in understanding the pathogenesis of high-risk *K. pneumoniae* strains.

Several methods have been established to classify *K. pneumoniae* strains. The most widely used method is multilocus sequence typing (MLST) based on the homology of seven genes, i.e. the *gapA*, *infB*, *mdh*, *pgi*, *phoE*, *rpoB* and *tonB* genes, in *K. pneumoniae* [7,8]. In this study, a term, sequence type (ST), is applied to describe classification results from the MLST method. Similar to the MLST method, a core-genome MLST (cgMLST) method identifies specific *K. pneumoniae* strains by evaluating the homology of over 2,000 genes conserved across *K. pneumoniae* genomes [9]. Alternatively, *K. pneumoniae* strains can be clustered based on the profile of core SNPs [10]. Geographical variations in CRKP strains are observed based on their MLST classification. A previous study [2] summarizes that ST258 is the most prevalent ST of CRKP in the Americas and Southern Europe, while ST11 is predominant in China. However, it is not clear whether or not certain STs have associations with the body sites of infections.

Previous methods classify *K. pneumoniae* based on a set of conserved housekeeping genes, providing broad phylogenetic resolution but lacking detailed functional insights on unique genes and biological functions in *K. pneumoniae* strains. Therefore, we aimed to investigate the genomic characteristics of CRKP strains using a novel approach based on centroid coding sequences (CDSs). Centroid CDSs are representative CDSs selected from clusters of highly similar CDSs (≥90% identity), extracted from complete genomes of *K. pneumoniae* strains, to reduce redundancy and capture the core genomic diversity of this species. This approach aims to address key questions about the genomic architecture of CRKP: (1) whether centroid CDSs can effectively capture genetic diversity among CRKP strains; (2) whether distinct STs harbour unique, functionally enriched centroid CDSs related to antimicrobial resistance and virulence; and (3) whether specific resistance and virulence genes significantly co-occur, which promotes hypothesis generation about potential interactions between resistance and virulence factors.

In this study, CRKP strains were classified using ST-associated centroid CDSs, revealing genetic diversity across STs. Several sets of centroid CDSs with enriched biological functions were observed in specific STs, contributing to pathogenicity and resistance. Additionally, significant coexistence of a specific set of antibiotic resistance and a group of virulence genes was identified in CRKP strains.

### Methods

### Collection of centroid CDSs on *K. pneumoniae* genomes

Complete genomes of 3,743 *K*. *pneumoniae* strains were downloaded from the NCBI genome database (https://www.ncbi.nlm.nih.gov/datasets/genome/) [11]. The accession numbers are listed in the Dataset S1 Sheet 1. Replicated genomes were removed using the ‘rmdup’ function in the seqkit software (version number: 2.10.0) with default parameters [12], which kept 3,592 unique genomes. CDSs were extracted from the genomes using the Prodigal software (version number: 2.6.3) with default parameters [13]. Subsequently, duplicated CDSs were deleted using the ‘rmdup’ function in the seqkit software. Finally, 47,222 centroid CDSs were selected using the ‘cluster_fast’ function in the vsearch software (version number: 2.30.0) with the parameter ‘--id’ set as ‘0.9’ and the rest of the parameters set as default [14]. The threshold to distinguish unique centroid CDSs was defined as 90% identity to be consistent with the similarity threshold used for the MLST classification [8].

### Annotation of centroid CDSs

nt sequences of the centroid CDSs extracted from the 3,592 *K*. *pneumoniae* complete genomes were annotated by alignment with the *K. pneumoniae* subsp. *pneumoniae* MGH 78578 transcriptome, the Comprehensive Antibiotic Resistance Database (CARD, version number: 3.3.0) [15] and the Virulence Factor Database (VFDB, downloaded on 28 August 2024) [16] using the ‘blastn’ function in the blast+ software (version number: 2.5.0) [17] with the following parameter: ‘-perc_identity 90 -qcov_hsp_perc 90 -evalue 1e-5 -max_target_seqs 1’ (Dataset S2). Additionally, the protein sequences of these centroid CDSs were aligned with the KEGG database using the hmmsearch software (version number: 3.3.2) with the hidden Markov model (default parameters with .HHM files as template) [18]. The four aforementioned methods assigned the centroid CDSs to genes on the reference genome, specific antibiotic genes, virulence genes and K numbers, respectively.

### Pretreatment of raw sequences of CRKP strains

There were 4,913 raw genome sequences of CRKP strains downloaded from the NCBI SRA database (https://www.ncbi.nlm.nih.gov/sra) [11] (see details, e.g. sequence ID, BioProject ID and source of isolation, in Dataset S1 Sheet 2). The total length of contigs from each sample was calculated, and samples with abnormal total contig lengths were removed in this study, which resulted in 4,458 samples. The ‘abnormal’ was determined by outlier detection using the ‘boxplot.stats’ function in R.

### MLST classification

The classification of MLST STs was determined as described in the previous publication [7,8]. Briefly, contigs belonging to each sample were aligned with a set of reference sequences downloaded from the BIGSdb database, involving those for the *gapA*, *infB*, *mdh*, *pgi*, *phoE*, *rpoB* and *tonB* genes, using the ‘blastn’ function in the blast+ software with the following parameter: ‘-perc_identity 90 -qcov_hsp_perc 90 -evalue 1e-5 -max_target_seqs 1’. The ST was determined based on the alignment results (Dataset S1 Sheet 2).

### Distribution of centroid CDSs in the genomes of CRKP strains

The distribution of centroid CDSs in the genomes of CRKP strains was tested by aligning the centroid CDSs to the contig pool described above using the ‘blastn’ function in the blast+ software with the following parameter: ‘-perc_identity 90 -qcov_hsp_perc 90 -evalue 1e-5 -max_target_seqs 50000’.

### Association between centroid CDSs and ST

The frequency of detection of a specific CDS across CRKP strains, categorized by belonging or not belonging to a particular ST, was quantified. The difference in CDS detection rates between these groups was assessed using the chi-squared test with the chisq.test() function in R (version number: 4.4.0) (Dataset S3). *P*-values from the chi-squared tests were adjusted using the Benjamini–Hochberg procedure.

### Clustering of CRKP strains

The CDSs that had been identified as significantly associated with any specific ST (Dataset S3) were involved in this analysis. The samples were clustered based on the presence of these ST-associated CDSs using the ‘pheatmap’ function in R with the following parameters: clustering_distance_cols = ‘binary’ and clustering_method = ‘ward.D2’. Since the presence or absence of a specific CDS was binary data, binary distance was calculated among samples. The Ward.D2 clustering method was chosen for its ability to minimize the total within-cluster variance, producing compact and interpretable clusters. Although originally developed for continuous data, Ward.D2 performs effectively on binary data when combined with an appropriate distance metric, e.g. binary distance [19]. Additionally, samples on the heatmap generated by the ‘pheatmap’ function were colour-coded by their matched ST and clustering results.

### Enrichment of K numbers in a specific ST

As described above, centroid CDSs were assigned to K numbers by aligning them with the KEGG database using the hmmsearch software. The distribution of these K numbers across the genomes of CRKP strains was determined based on the distribution of the corresponding matched CDSs. Over-representation analysis was performed using the phyper() function in R to identify K numbers with larger copy numbers of matched CDSs in CRKP strains belonging to a specific ST, compared with all CRKP strains (Dataset S4). *P*-values from the over-representation analysis were adjusted using the Benjamini–Hochberg procedure. Adjusted enrichment of a specific K number in a given ST was calculated by comparing the detection frequency of centroid CDSs assigned to that K number in CRKP strains of the specific ST with the detection frequency of the same K number across all genomes, normalized by the median detection frequencies of all K numbers within the ST and across all STs.

### Enrichment of antibiotic and virulence genes in a specific ST

As described above, the CDSs were assigned to antibiotic and virulence genes by alignment with the CARD and VFDB databases, respectively. The enrichments of antibiotic and virulence genes in a specific ST were performed using the same method as introduced in the enrichment of K numbers in a specific ST using the over-representation analysis.

### Coexistence of antibiotic resistance and virulence genes

The presence and absence of a specific gene in a genome were coded as 1 and 0, respectively. The coexistence of an antibiotic gene and a virulence gene was tested using Pearson’s correlation. A positive *R*-value closer to 1 indicates a higher rate of coexistence between the two genes. *P*-values from Pearson’s correlation test were adjusted using the Benjamini–Hochberg procedure. Significantly and strongly co-existed antibiotic and virulence gene dyads [False Discovery Rate (FDR) ≤0.05 and *R*≥0.7] were input to the Gephi software for the visualization of the coexistence network. Alternatively, Fisher’s exact test was performed to validate the *P*-values obtained from Pearson’s correlation analysis.

## Results

### Centroid CDSs in the genomes of *K. pneumoniae*

**Fig. 1.**
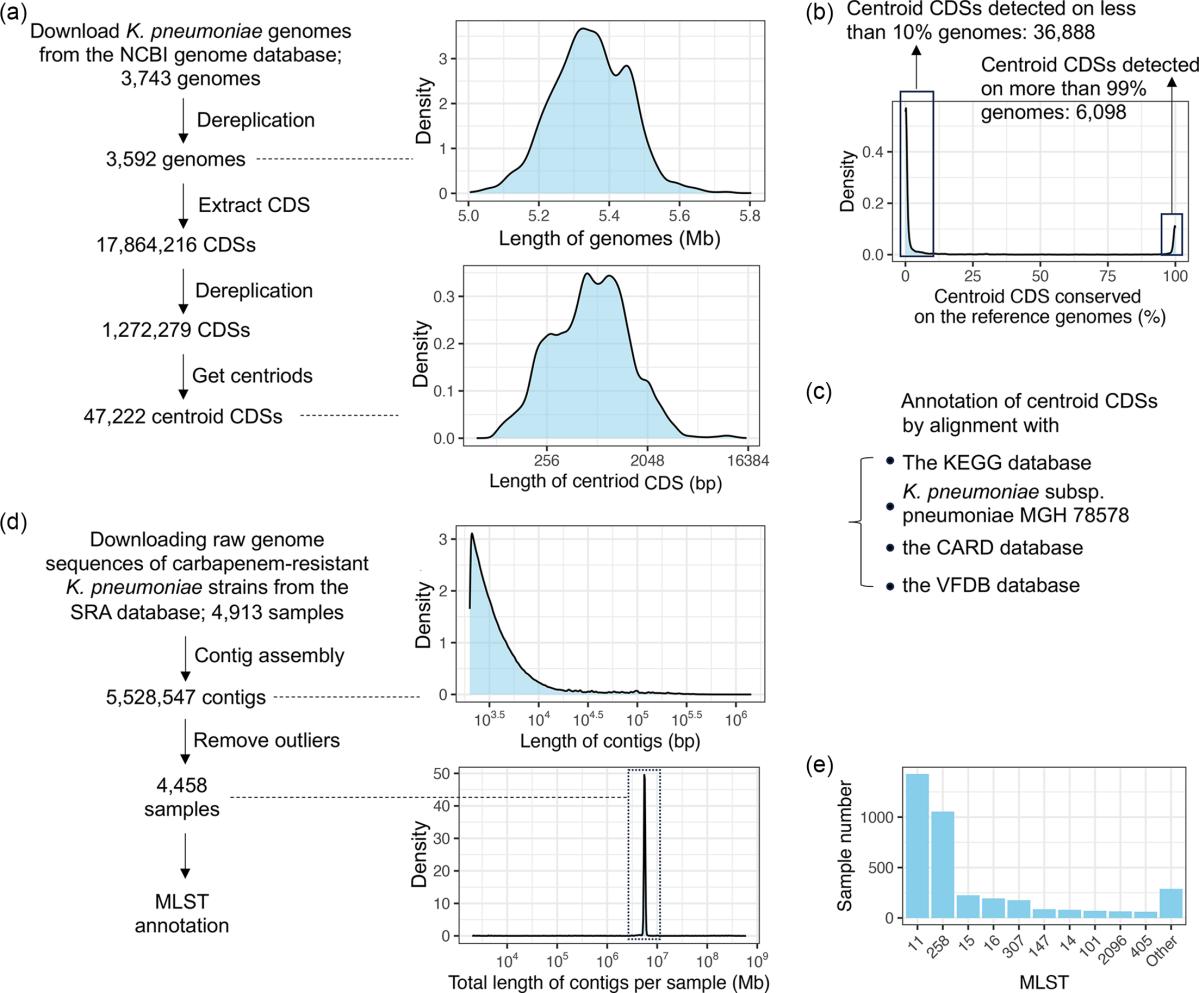
Collection and analysis of centroid CDSs and contigs from CRKP strain data. (**a**) Illustration of methods for collecting centroid CDSs from complete genomes of *K. pneumoniae* strains, along with the length distribution of complete genomes and centroid CDSs. (**b**) Visualization of the conservation distribution of these centroid CDSs within complete genomes of *K. pneumoniae* strains. (**c**) Methods for annotating centroid CDSs. (**d**) Procedures for collecting contigs from raw sequencing data for CRKP strains. (**e**) Classification of CRKP strains using the MLST method. More details about the complete genomes and raw sequencing data for CRKP strains are listed in Dataset S1, and annotations for the centroid CDSs are listed in Dataset S2.

**Fig. 2.**
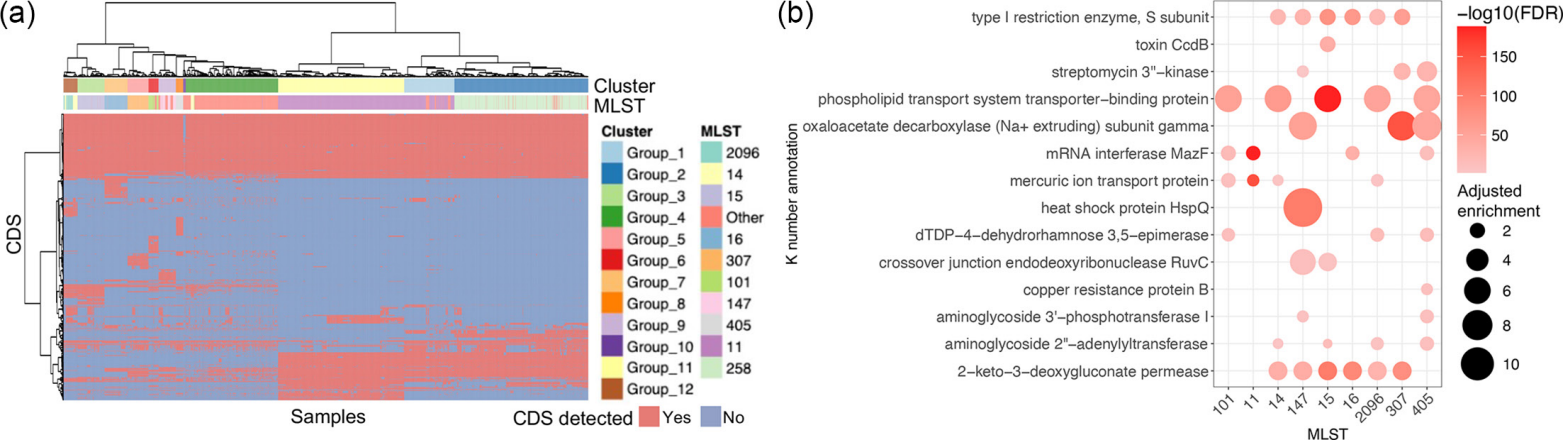
Association between MLST and centroid CDS methods in CRKP strains. The association between centroid CDSs and STs was tested (see Methods). (**a**) CRKP strains were classified based on the presence of MLST-associated centroid CDSs. Binary distances among strains were calculated, and hierarchical clustering was performed using the ‘ward.D2’ method. The x-axis and y-axis represent the clustering of centroid CDSs and CRKP strains, respectively. CRKP strains are colour-coded according to clustering outcomes and MLST types. (**b**) Centroid CDSs were assigned to K numbers, and over-representation analysis was conducted to identify K numbers with higher copy numbers of matched CDSs in specific ST groups compared with all CRKP strains. The biological functions of enriched K numbers based on KEGG annotations are shown.

After dereplication, there were 3,592 complete genomes of *K. pneumoniae* downloaded from the NCBI genome database with the distribution of genome lengths between 5 and 5.8 Mb (Fig. 1a). Forty-seven thousand two hundred twenty-two centroid CDSs were extracted from these genomes as described in Methods. To be consistent with the similarity threshold used for the ST classification [8], the threshold to distinguish unique CDSs was defined as 90% identity. The majority of CDSs were between 100 and 3,000 bp (Fig. 1a). The conservation of the centroid CDSs across the 3,592 reference genomes was tested based on the nt sequence alignment of these CDSs with the reference genomes with the identity threshold set as 90%. About 13% (6,098) of the centroid CDSs were highly conserved in more than 99% of the reference genomes (Fig. 2b). In contrast, most CDSs (78% or 36,888) were unique with detection rates lower than 10%. Therefore, specific characteristics associated with the genomes of *K. pneumoniae* could be examined by the presence or copy number of the set of unique CDSs.

To understand the biological meanings of the centroid CDSs, sequence alignments were performed to compare these CDSs against reference sequences in four databases, i.e. the KEGG database, reference transcriptome of *K. pneumoniae* subsp. *pneumoniae* MGH 78578, the CARD database and the VFDB database (see Methods). There were 23,662, 7,858, 118 and 389 CDSs assigned to specific K numbers, genes on the genome of *K. pneumoniae* subsp. *pneumoniae* MGH 78578, antibiotic resistance genes and virulence factor genes, respectively (Dataset S2). More details are shown below.

### Classification of CRKP strains by MLST

As described in Methods, raw sequencing data for CRKP strains were obtained from the NCBI SRA database, with the corresponding metadata provided in Dataset S1 Sheet 2. Each sample indicates a CRKP strain. After assembling contigs for each sample, those with exceptionally low or high total contig lengths, identified through outlier analysis, were excluded. Low contig lengths suggested insufficient sequencing depth, while high values indicated potential contamination in sequencing (Fig. 1d). A total of 4,458 samples with contig lengths between 5 and 6 Mb were assigned to STs (see Methods). This large dataset is crucial for uncovering genomic differences, as it enhances statistical power and enables the detection of rare strain types and patterns that smaller datasets might overlook. Consistent with previous studies, STs 11 and 258 were the most frequently identified types for these CRKP strains [20], followed by STs 15, 16, 307, 147, 14, 101, 2096 and 405 and other less common types (Fig. 1e).

### Classification of CRKP strains by unique centroid CDSs

The previously reported methods, e.g. ST [7] and cgMLST [9], classify *K. pneumoniae* strains based on homology among a set of genes conserved across all strains. Here, we provide an alternative way to classify the *K. pneumoniae* strains based on the ST method and the detection of the presence of centroid CDSs.

Initially, the association between each centroid CDS and each ST was assessed using a chi-squared test to determine whether a specific CDS exhibited a significantly different detection rate in *K. pneumoniae* strains of a particular ST compared to all strains. Hence, 4,789 centroid CDSs were found to be associated with at least one ST (Dataset S3). Subsequently, the samples were clustered into 12 groups using the ‘ward.D2’ clustering method, based on binary distance calculations derived from the 4,789 ST-associated centroid CDSs (Fig. 2a). Interestingly, most samples with the same ST were grouped together. For instance, most samples of STs 258, 15, 16 and 307 were classified into groups 2, 3, 7 and 5, respectively. A Pearson’s chi-squared test further demonstrated a strong association between the classification outcomes of the MLST and centroid CDS methods, with a *P*-value less than 2.2e-16.

Samples with ST11 were divided into two groups, i.e. groups 1 and 11, implying the presence of two subgroups of ST11 strains based on the presence of ST-associated centroid CDSs. Other rarely detected STs were also assigned to different groups. For example, samples with STs 340, 395 and 437 were classified into group 1 alongside ST11 (Dataset S1 Sheet 2 columns G and H), implying that strains with these STs may share genomic similarities with ST11, particularly in terms of unique gene composition. However, it is worth noting that the formation of these groups was based on only 4,789 ST-associated centroid CDSs, rather than the full set of 47,222 centroid CDSs, which may introduce potential technical variability.

### Enriched biological functions in specific MLSTs

The substantial overlap between the classification results from the ST method and the centroid CDS method (Fig. 2a) indicated that strains within each ST possessed a distinct set of centroid CDSs in their genomes. To gain deeper insight into the biological implications of the ST classification method, the centroid CDSs were mapped to corresponding K numbers by aligning them with the KEGG database (see Methods). An over-representation analysis was subsequently performed to identify K numbers with significantly higher copy numbers on genomes of specific STs (Fig. 2b and Dataset S4).

The S subunit of the type I restriction enzyme is enriched in STs 14, 147, 15, 16, 2096 and 307, while the toxin CcdB is abundant in ST15 (Fig. 2b). Type I restriction-modification systems in *K. pneumoniae* can degrade exogenous DNA, e.g. blaKPC genes, reducing antibiotic resistance [21,22]. Meanwhile, CcdB, a protein involved in plasmid maintenance, has been reported to support the persistence of *blaNDM* and *blaCTX-M* plasmids in *Escherichia coli* under carbapenem and cephalosporin pressure [23]. The enrichment of the S subunit of the type I restriction enzyme and the ccdB gene in specific STs is therefore noteworthy, as it may reflect underlying genomic features associated with resistance phenotypes. However, these findings are based on genomic association analyses and do not imply a direct functional or causal relationship. Further experimental studies are needed to elucidate the mechanistic roles of these genes. Details of the relevant CDSs are listed in Dataset S2, with matched sequences available on GitHub (https://github.com/crkp-lgz/crkp).

Four sets of proteins, i.e. streptomycin 3′-kinase, dTDP−4−dehydrorhamnose 3,5−epimerase, aminoglycoside 3′-phosphotransferase I and aminoglycoside 2′-adenylyltransferase, contribute to resistance against aminoglycoside antibiotics. One or more of these proteins are enriched in STs 147, 307, 405, 15 and 2096 (Fig. 2b), suggesting a potential association between aminoglycoside and carbapenem resistance in these STs. Potential hypotheses for this association are explored below. Similarly, the phospholipid transport system has been associated with resistance to polymyxin B, a ‘last-resort’ antibiotic for CRKP [24], and is enriched in STs 101, 14, 15, 2096 and 405.

Mercuric ion transport and copper resistance proteins are linked to metal ion resistance. Mercuric ions are toxic to *K. pneumoniae* but can stimulate biofilm formation at certain concentrations [25]. The roles of MazF, HspQ, RuvC and 2-keto-3-deoxygluconate permease in carbapenem resistance in *K. pneumoniae* remain unclear.

### Coexistence of antibiotic and virulence genes

A total of 118 and 389 CDSs were identified as antibiotic resistance genes and genes encoding virulence factors, respectively (Dataset S2). Over-representation analysis illustrated the enrichment of distinct antibiotic resistance and virulence genes across various STs (Fig. S1), further demonstrating the genomic diversity within these STs.

**Fig. 3.**
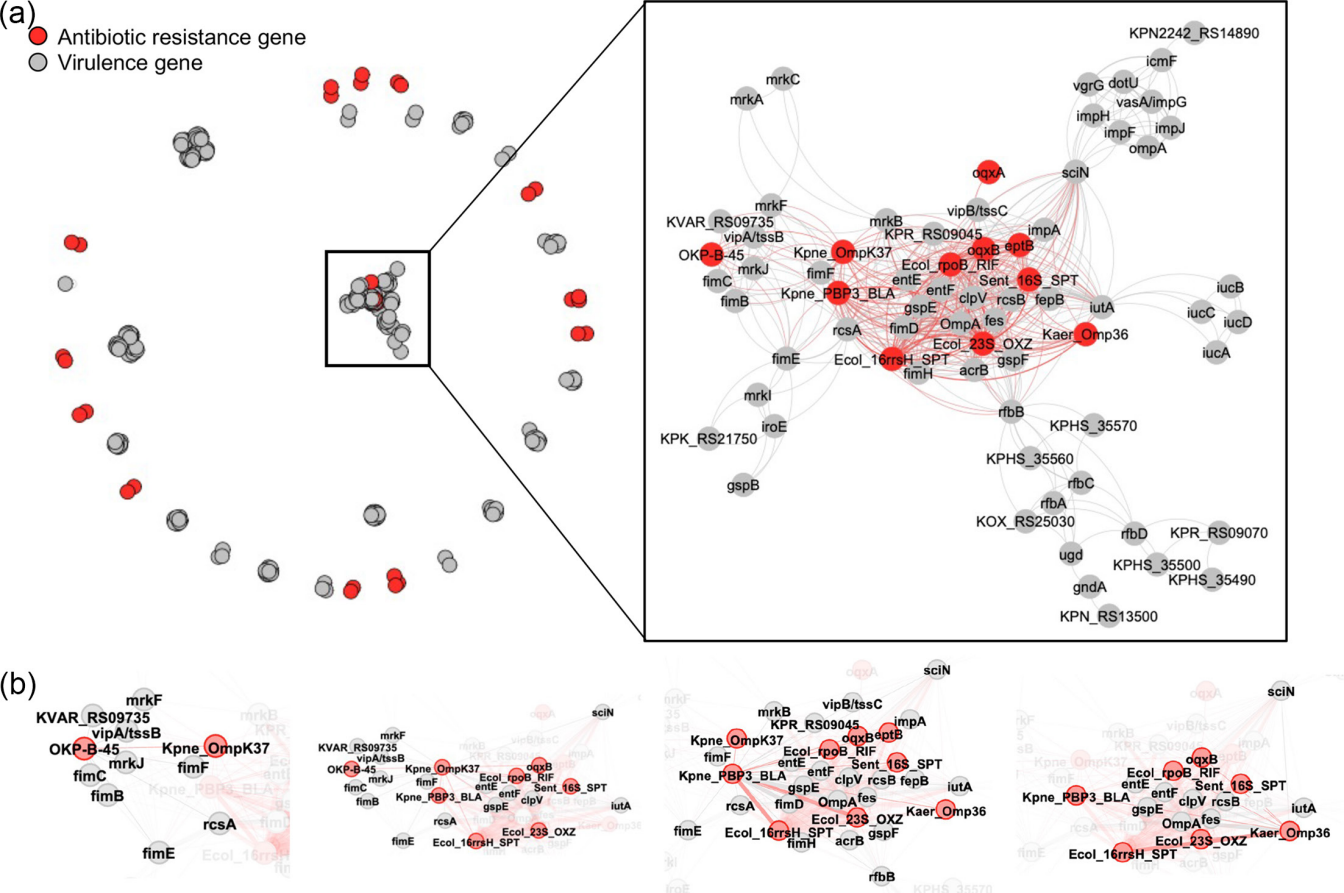
Coexistence of antibiotic resistance and virulence genes in CRKP strains. Centroid CDSs were assigned to antibiotic resistance and virulence genes using the CARD and VFDB databases, respectively. Coexistence among antibiotic resistance and virulence genes was assessed using Pearson’s correlation, with *P*-values adjusted by the Benjamini–Hochberg procedure. Gene dyads with significant and strong coexistence (FDR≤0.05 and *R*≥0.7) were visualized using Gephi software. (**a**) Network representation of significant gene coexistence. (**b**) Significant coexistence relationships for each carbapenem resistance gene are shown. Information for CDSs assigned to carbapenem resistance genes without significant coexistence with virulence genes can be found in Dataset S2.

Coexistence analysis was conducted using two methods, i.e. Pearson’s correlation and Fisher’s exact testDataset S5; see Methods for details). Based on the correlation results, a network was constructed, revealing that more than half of the identified antibiotic resistance genes were not significantly associated with virulence genes (Fig. 3a). However, a group of carbapenem resistance genes, along with several aminoglycoside, peptide and tetracycline resistance genes, significantly coexisted with virulence genes (right panel of Fig. 3a and Dataset S2). The coexistences between four carbapenem resistance genes and other genes in the coexistence network are visualized in Fig. 3(b).

Among the virulence genes significantly coexisting with antibiotic resistance genes (Fig. 3), those encoding type I and III fimbriae (*fimB*, *fimE*, *fimC*, *fimD*, *fimF*, *fimH*, *mrkC*, *mrkI*, *mrkJ*, *mrkF*, *mrkB* and *mrkA*) have been linked to increased biofilm formation, a key factor in antibiotic resistance in *K. pneumoniae* [26]. Similarly, genes involved in efflux pumps (*impA*, *impF*, *impH* and acrB) enhance antibiotic resistance by expelling drugs [27]. Additionally, the two-component system genes *rcsA* and *rcsB* modulate antibiotic resistance under stress [28]. However, the role of the type II secretion system (*gspB*, *gspE* and *gspF*) and siderophore-mediated iron acquisition (*fes*, *entE*, *fepB* and *entF*) in antibiotic resistance of *K. pneumoniae* remains unclear.

Consistent with the functional enrichment analysis described above, carbapenem resistance genes, i.e. *ompK37* (encoding outer membrane porin 37, shown as Kpne_OmpK37 in Fig. 3), a gene encoding *β*-lactamase-associated penicillin-binding protein 3 (Kpne_PBP3_BLA) and *omp36* (Kaer_Omp36), coexisted with genes for aminoglycoside resistance, i.e. Ecol_16rrsH_SPT, Sent_16S_SPT and Ecol_23S_OXZ (see detailed annotations in Dataset S2), in CRKP strains (Fig. 3b). However, whether aminoglycoside and carbapenem resistance coexist requires further validation in clinical settings.

### Distribution of STs across countries and body sites

**Fig. 4.**
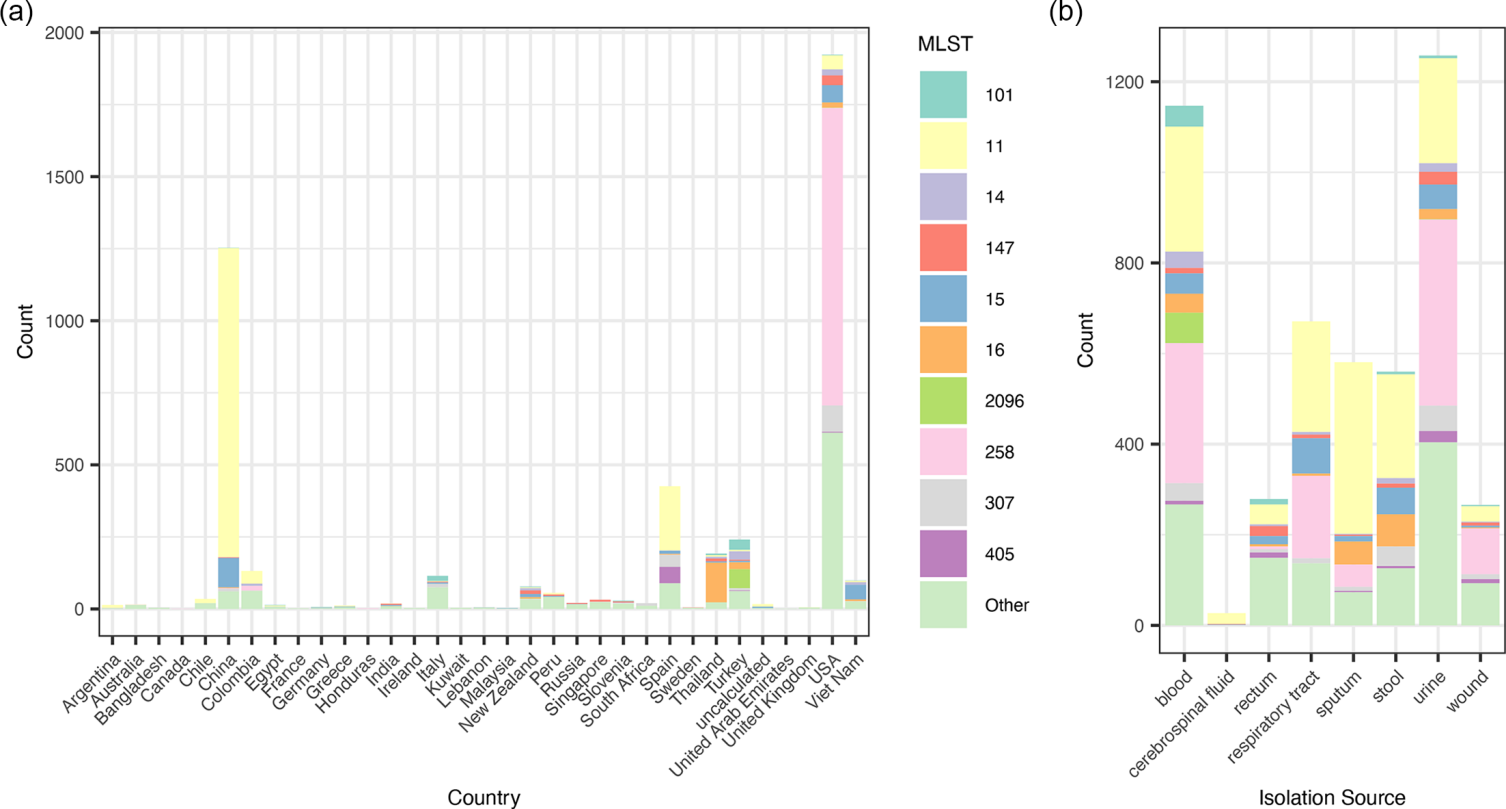
Distribution of STs of CRKP strains. The distribution of STs of CRKP strains categorized by country (**a**) and body site of isolation (**b**) is shown.

As shown in Fig. 4(a), each country appears to have several predominant STs specific to its CRKP strains. For example, the major STs in China are ST11, followed by ST15, while in the USA, Spain, Thailand and Vietnam, the predominant STs are ST258, ST11, ST16 and ST15, respectively. In contrast, no specific ST is enriched in any of the studied body sites (Fig. 4b). Therefore, an enrichment analysis using the over-representation test to identify biological functions associated with specific body sites yielded few significant results (data not shown). Additionally, because some sample types, e.g. cerebrospinal fluid, were primarily from one country, it was unable to determine whether the detected association was determined by the country factor.

## Discussion

Previous methods, i.e. MLST and cgMLST, classify CRKP strains based on homologous sets of conserved genes [7,10]. This study proposed an alternative approach that groups CRKP strains by the presence of MLST-associated centroid CDSs on the genome. While MLST and cgMLST highlight phylogenetic differences, the centroid CDS strategy emphasizes unique genomic features. Notably, clustering results from the centroid CDS approach largely overlapped with those of MLST, suggesting strong consistency between the methods. Moreover, the centroid CDS method offers additional biological insights into the MLST outcomes, as demonstrated in Fig. 2(b). Overall, this method offers a complementary perspective, enhancing understanding of genomic composition and its biological implications.

In our analysis pipeline, contig assembly was employed instead of assembling complete genomes. This approach offers several advantages: (1) CDSs with matched genes not characterized in previous studies can be included in the analysis; (2) it allows the inclusion of more samples, as a complete genome is not mandatory; (3) it simplifies the analysis process by eliminating the need for genome assembly. However, to maintain data quality, a threshold for total contig length was set to filter out low-quality samples (see Methods). Therefore, sufficiently deep sequencing remains essential for this pipeline.

It is important to note that this method uses CDSs instead of genes, leading to multiple centroid CDSs being matched with the same gene. As a result, interpreting the biological significance of centroid CDSs is more complex than with the more direct MLST or cgMLST methods. Additionally, the contig assembly was based on raw sequencing data, which could originate from either genomic or plasmid DNA. As a result, it is unable to distinguish genomic CDSs from plasmid CDSs. To mitigate this problem, whole-genome sequencing is necessary to assemble complete plasmid and genome sequences, enabling accurate identification of the origin of each CDS.

As shown in the results, coexistence between genes for carbapenem and aminoglycoside resistance was identified. This phenomenon could be explained by the coexistence of these genes on the same plasmids or co-transmission of these genes from plasmid to the genome of *K. pneumoniae* [29]. Alternatively, resistant strains, especially those carrying multiple resistance genes, may have a selective advantage in environments where both aminoglycosides and carbapenems are used. Furthermore, all findings regarding the enrichment of biological functions in specific MLSTs and the coexistence of antibiotic and virulence genes reflect genomic-level associations or correlations. Whether these relationships are causative requires further experimental and clinical validations.

Although no significant association was observed between MLST classifications and body sites, potential biases in sample selection, e.g. the uneven geographic distribution of samples, predominantly from the USA and China, may have influenced the observed patterns of gene coexistence and functional enrichment. As more genomic sequencing data become available, future studies should incorporate more sophisticated statistical models to account for these potential confounding factors and improve the robustness of the analyses.

In conclusion, this study introduced a novel method for classifying *K. pneumoniae* strains using MLST-associated centroid CDSs, revealing unique genomic features and enriched functions in specific STs. This method complements traditional approaches, enhancing the understanding of CRKP strain diversity and resistance mechanisms.

## Supplementary material

10.1099/mgen.0.001457 Uncited Fig. S1.

10.1099/mgen.0.001457 Uncited Supplementary Data Sheet 1.

10.1099/mgen.0.001457 Uncited Supplementary Data Sheet 2.

10.1099/mgen.0.001457 Uncited Supplementary Data Sheet 3.

10.1099/mgen.0.001457 Uncited Supplementary Data Sheet 4.

10.1099/mgen.0.001457 Uncited Supplementary Data Sheet 5.

## Abbreviations

CARD: Comprehensive Antibiotic Resistance Database
CDS: coding sequence
cgMLST: core-genome MLST
CRKP: carbapenem-resistant *Klebsiella pneumoniae*
FDR: false discovery rate
MLST: multilocus sequence typing
ST: sequence type
VFDB: Virulence Factor Database
