# Supplementary material for: Genomic diversity and functional insights of carbapenem-resistant Klebsiella pneumoniae revealed by centroid coding sequences analysis
Source: Microb Genom. 2025 Jul 25;11(7):001457. doi: 10.1099/mgen.0.001457 (PMC12452191; doi:10.1099/mgen.0.001457)
Supplement: Uncited Fig. S1. [file mgen-11-01457-s001.pdf]

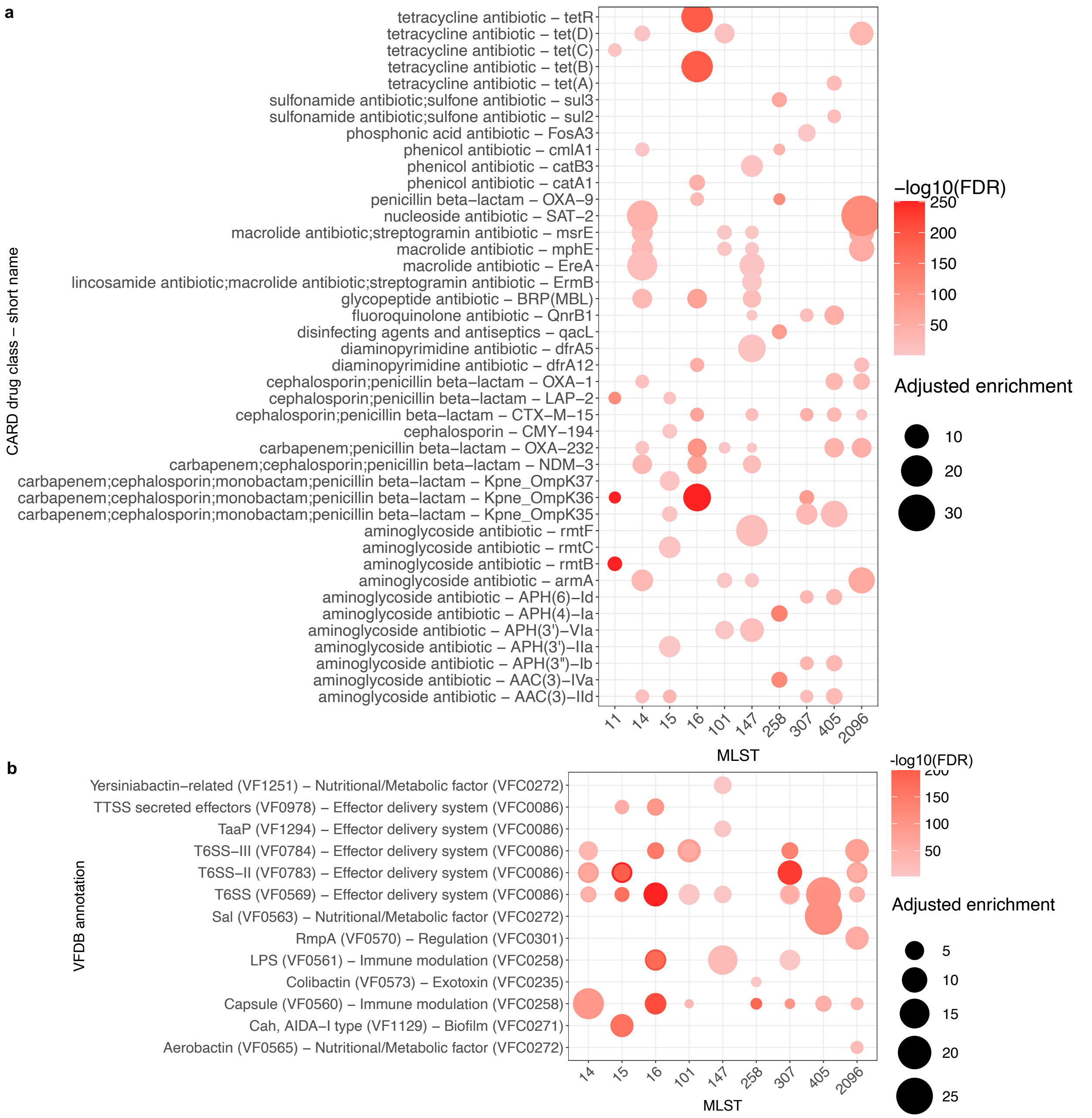

Fig. S1 Association between MLST and carbapenem resistance and virulence genes in CRKP strains. Centroid CDSs were assigned to carbapenem resistance **(a)** and virulence genes **(b)**, and over-representation analysis was conducted to identify these genes with higher copy numbers of matched CDSs in specific ST groups compared to all CRKP strains.
